# Modulatory immune responses in fungal infection associated with organ transplant - advancements, management, and challenges

**DOI:** 10.3389/fimmu.2023.1292625

**Published:** 2023-12-07

**Authors:** Amir Elalouf, Hadas Elalouf, Ariel Rosenfeld

**Affiliations:** ^1^ Department of Management, Bar-Ilan University, Ramat Gan, Israel; ^2^ Information Science Department, Bar-Ilan University, Ramat Gan, Israel

**Keywords:** fungal infections, organ transplantation, immune response, management - healthcare, challenge

## Abstract

Organ transplantation stands as a pivotal achievement in modern medicine, offering hope to individuals with end-stage organ diseases. Advancements in immunology led to improved organ transplant survival through the development of immunosuppressants, but this heightened susceptibility to fungal infections with nonspecific symptoms in recipients. This review aims to establish an intricate balance between immune responses and fungal infections in organ transplant recipients. It explores the fundamental immune mechanisms, recent advances in immune response dynamics, and strategies for immune modulation, encompassing responses to fungal infections, immunomodulatory approaches, diagnostics, treatment challenges, and management. Early diagnosis of fungal infections in transplant patients is emphasized with the understanding that innate immune responses could potentially reduce immunosuppression and promise efficient and safe immuno-modulating treatments. Advances in fungal research and genetic influences on immune-fungal interactions are underscored, as well as the potential of single-cell technologies integrated with machine learning for biomarker discovery. This review provides a snapshot of the complex interplay between immune responses and fungal infections in organ transplantation and underscores key research directions.

## Introduction

Significant breakthroughs in organ transplantation are one of the key advancements of modern science. Organ transplantation is presently the most sustainable and cost-effective therapeutic option for end-stage organ diseases and failure, thereby most likely the only chance for the patient’s survival. The continuous struggle to decipher immune responses upon transplantation and infection has been pivotal in the clinical application of organ transplantation (1). Milestones in the field of transplantation have been achieved through a long-convoluted path, from its inception in mythology to gradual reality. Initial accounts of organ transplants of bone, skin, and heart can be traced back to ancient mythologies of Greece, China, and Rome. In 1550 BC, there were reports of a historical attempt of skin grafts to treat burns (2), which led to the first human kidney transplant in 1933 by a Ukrainian surgeon U.U. Voronoy. Due to an insufficient understanding of the immune system, the transplanted kidney could not produce urine, and the patient survived for only two days (3). In 1954, the American surgeon Murray performed a kidney transplant in monozygotic twins, where the recipient survived for eight years with normal kidney function (4).

Subsequently, advancements in immunology played a vital role in developing immunosuppressants, which greatly improved the survival of organ transplants. AZA (Azathioprine) was developed first to inhibit lymphocyte proliferation by inhibiting DNA, RNA, and protein synthesis via purine antagonism. Consequently, AZA was an immunosuppressive drug to lower organ rejection for the first successful cadaveric kidney transplant (5, 6). CsA (Cyclosporine A) was synergistically used with glucocorticoid and showed promising results in raising the survival of recipients to one year, particularly for kidneys (95%) and liver (75%) transplants (7–9). Consequently, other immunosuppressive drugs were approved for liver-kidney and kidney transplantation, including FK506 (Tacrolimus) and Rapamycin, which demonstrated promising therapeutic potential with improved recipient tolerance (10).

Even nowadays, the main challenge in organ transplantation is the increasing demand for organ transplants, which outstrips the supply. Several strategies have been explored to tackle this issue, including extended criteria grafts, machine perfusion for organ preservation of initially inferior quality, living donors, and bioprinting (11–13). Another path rapidly explored is the creation of organoids mimicking solid organs in regenerative medicine, which is still evolving (14, 15). Xenotransplantation emerged as a promising field to reduce the waitlist for organ transplants. In 1964, Dr. Reemtsma was able to successfully xenotransplant a baboon kidney, where the patient survived for nine months with immunosuppression drugs (16). Xenotransplantation is presently the leading technology that underwent a qualitative leap in 2013 with CRISPR/Cas9 genome editing technology. CRISPR/Cas9 has enabled multiple, efficient modifications in the animal genome to overcome rejection and facilitate immune and coagulation processes in recipients. Challenges associated with xenograft rejection include standardizing predictive markers such as CD3, CD4, and CD8 and those related to cellular injury (17). The report for the first clinical trial has demonstrated that specific immunological routes should be developed apart from gene editing tools to ensure xenotransplantation is achieved (18). The outcome of further human clinical trials using xenotransplants will soon shed light on selecting recipients for a xenotransplant and elucidate optimal immunosuppression regimens for tolerance and long-term survival.

The success of organ transplantation was intrinsically woven with advances in understanding the immune responses at the molecular and cellular levels. The application of immunosuppressant drugs improved organ rejection and optimized patient survival. Due to the imposition of immunosuppressive regimens, organ recipient exhibits heightened vulnerability to fungal infections during the initial six months following transplantation. Systemic fungal infections in these recipients manifest with nonspecific clinical symptoms. Timely detection of fungal infections is imperative to ensure appropriate therapeutic interventions, enhancing patient survival rates and mitigating mortality. The most frequently documented fungal agents include *Candida* spp. and *Cryptococcus* spp., while filamentous fungi notably comprise *Aspergillus spp* (19). Within the human host, certain dimorphic fungi, such as *Coccidioides posadasii, Coccidioides immitis, Blastomyces dermatitidis*, and *Histoplasma capsulatum*, exist predominantly in the yeast form (20). Among organ transplant recipients, histoplasmosis accounts for approximately 5% to 9% of fungal infections, with the occurrence among kidney transplant recipients after 2 to 5 years post-transplantation estimated at 0.1% to 0.3% (1, 21–23).

This review aims to present a comprehensive and in-depth analysis of the intricate interplay between immune responses and fungal infections observed in organ transplantations. The study systematically investigates the fundamental immunological mechanisms governing infections, highlights recent advancements in our comprehension of immune response dynamics, and conducts a rigorous evaluation of strategies employed for immune modulation. The review mainly addresses the mechanism of immune responses against fungal infection in organ transplantation, along with immunomodulatory approaches, diagnostic methods, treatment modalities, challenges, and management strategies.

## Immune responses in organ transplant

### Immune system in organ transplant- modulation, signaling, and activation

**Figure 1:**
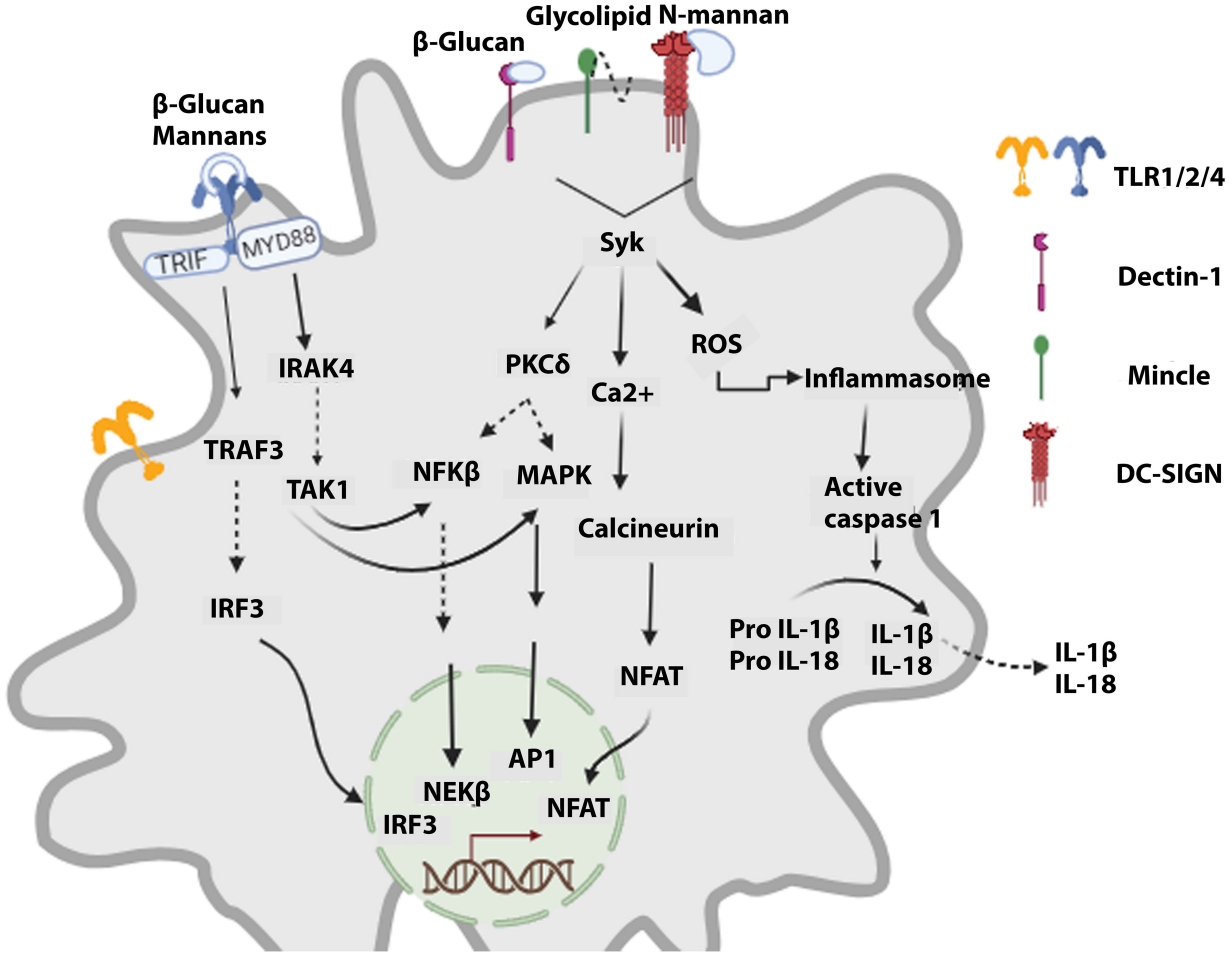
Signaling cascades activated in innate cells upon recognizing fungal components. Fungal PAMPs lead to the signaling of TLRs via MyD88, which orchestrates the activation of NFKβ and MAPKs, resulting in antifungal action. CLRs (dectin-1, DC-SIGN, Mincle) via Syk activate the calcineurin-NFAT signaling cascade and ROS (reactive oxygen species) production. Furthermore, it leads to inflammasome activation, which promotes maturating of caspase 1. Caspase 1 induces the generation of active forms of cytokines IL-1β and IL-18, which are then released.

The immune system’s role is to detect, protect, and destroy foreign invaders and abnormal cells. The immune system consists of a complement system and innate and adaptive immunity. In the immune response reaction, innate kick-starts within 24 hours after transplantation (24). Immune responses result in organ rejection, where innate and adaptive immune cells employ different cascades to reject the transplant. The innate immune system identifies PAMPs (pathogen-associated molecular patterns) and DAMPs (damage-associated molecular patterns) of antigens as non-self leads to immune system activation. PAMPs are conserved components distinctive to microbes and are absent in the human body. APCs (Antigen-presenting cells) have unique PRRs (pathogen recognition receptors) that, upon binding PAMPs, lead to events that stimulate cytokine release and activate the complement system, destroying the pathogen via phagocytosis (**Figure 1**).

Surgical trauma and IRI (ischemic-reperfusion injury) are events in organ transplantation associated with tissue damage whereby IRI cells in the donor organ undergo apoptosis, necrosis, and ferroptosis, leading to the release of DAMPs (25–27). During IRI and apoptosis, HSP (heat shock proteins), HMGB-1 (high mobility group box protein-1), and hyaluronan are secreted (25, 28), bind to TLR, which activate the dendritic cells (DCs), release of cytokines, and recruitment of other immune cells, like macrophages and neutrophils at the site of inflammation (29). This reaction leads to high inflammation at the site of organ transplant (26, 29). In lung transplant, it has been shown that neutrophils infiltrated the organ upon transplantation and interacted for 7 minutes with donor graft resident DCs, whereby the donor DCs possibly upregulated IL-2 expression and MHCII (Major histocompatibility complex II) (24). Danger signals also contribute to another phenomenon called trained immunity, which corresponds to memory responses and plays a significant role in the outcome of organ transplants. Allograft infiltrating macrophages express TLP-4 and dectin-1 and recognize HMGB-1 and vimentin, respectively. Apoptotic cells’ surfaces have vimentin, which trains the infiltrated macrophages upon recognition. When receiving another stimulus, these trained macrophages release high pro-inflammatory TNF-α and IL-6 cytokines, resulting in allograft rejection (30). These trained macrophage responses have been shown to last for months after the initial stimulus terminates (30, 31). Inhibiting trained immunity via short-term mTORi-HDL immunotherapy promoted organ transplant acceptance (31). Innate immune cells also recognize allo and xeno-antigens, as in a preliminary study, macrophages rejected xenografts without T and B lymphocytes (32). Neutrophils invade xenografts (cellular and organ) (33, 34) and lead to a release of neutrophil extracellular traps and production of ROS (reactive oxygen species) and digestive enzymes (35, 36). Xenograft rejection by NK (natural killer) cells is seen via a receptor [e.g., natural killer group-2D (NKG2D)] and porcine UL16-binding protein-1 (pULBP-1) (37), which upon binding result in perforin and granzymes release (38). The recognition of SLA-1 and -2 complexes by T cell receptors initiates adaptive immunity against the xenograft (39), whereas CD4+ T cells are recruited when xenograft antigens are presented by the recipient APCs, leading to antibody-mediated rejection (39). Consequently, the release of cytokines in this process further induces immune responses by NK cells and macrophages (40). B cells also contribute to xenograft rejection as reports have shown that the transplant of heart from pig to baboon showed 8-month survival when the B cells were removed, hence also reiterating that depletion of B cells can delay xenograft rejection (41). The production of anti-Gal antibodies by B cells binds to Gal antigens in porcine tissues, inducing rejection, whereas the removal of anti-Gal antibodies hampers the rejection of xenograft (42, 43).

Studies have shown distinctions between danger and allogenic signal-mediated innate immunity activation. Recipient’s monocyte upon allogenic signaling helps to monocyte differentiation into mature DCs, whereas danger signals direct to less mature DCs. Fully mature mono-DCs will collude with the adaptive immune system (44), orchestrated and amplified by innate immunity in many ways. The activated DCs trigger the adaptive immune system, which travels to the recipient’s lymphoid tissue, expressing alloantigen bound to MHC. Donor naïve T cells recognize MHC-bound alloantigen, resulting in the proliferation and differentiation of T cells. Upon reaching the graft site, Cytotoxic T cells will identify the host antigen and lead to graft rejection. Some T cells survive as memory cells and remain a liability to the transplant’s short and long-term survival (45).

After organ transplantation, early acute T cell-mediated rejection is governed by direct allorecognition; the donor APCs with MHC-I and MHC-II of alloantigens bind to naïve CD8 and CD4 cell receptors, resulting in activation and differentiation. Upon reaching the transplant site, the primed CD8 cytotoxic cells identify the allogenic MHC molecules, causing cell damage and even tissue rejection in extreme cases (46). Indirect allorecognition mediates late acute T cell-mediated rejection. The APCs of the recipient take donor allogeneic antigens and present them to T cells through self-MHC molecules. In this case, as for the direct pathway, T cells activate, proliferate, and differentiate with long-term immune response (47). Recurring events of T cell-mediated rejection can lead to chronic T cell-mediated rejection, where memory T cells at a low activation threshold can lead to graft rejection (48), along with other contributory factors such as repeat transplantation, tacrolimus (FK-506)-free immunosuppression regimen (46).

Regulatory T cells (Tregs) are lymphocytes that control the activity of other immune cells and are essential in the initiation and conservation of peripheral tolerance, hence maintaining a check on immune responses and autoimmunity. Tregs cause cell apoptosis by releasing IL-10, TGFβ, IL-35, and perforin. In an indirect mechanism, Tregs, using CD39/CD73, reduce the presence of extracellular ATP in the surrounding area, which leads to immunosuppression (49). High expression of the CD25 receptor induces uptake of IL-2, depleting the availability of this cytokine for the other cells (50). Tregs in the graft microenvironment contribute significantly to the induction of graft tolerance. In transplantation, CD25^+^CD4^+^FOXP3^+^ Tregs mainly contribute to managing immune responses to alloantigens and suppressing graft rejection (51). *In vivo* (52) and ex vivo (53) exposure to alloantigen-induced CD25^+^CD4^+^FOXP3^+^ Tregs were similar to Tregs derived from the thymus. In a graft recipient, donor alloantigen reactive Tregs can be formed and demonstrate various routes of action. The hypothesis suggesting that the adoptive transfer of Tregs can both decrease graft rejection and promote graft tolerance in a new environment has been evaluated in preclinical animal models. Encouraging outcomes were achieved through the infusion of Tregs (54–56). Preclinical data illustrated that the alloantigen-specific Tregs can be more effective (57). The development of Tregs with chimeric antigen receptors provides the required specificity (58, 59) where these Tregs in animal models were able to reach the graft (60, MacDonald et al., 2016). These Tregs suppressed skin graft rejection considerably compared to polyclonal Tregs (61, 62).

B-lymphocytes on their surfaces express antigen-specific receptors called immunoglobulins. B cells are activated when these receptors bind to HLA antigens of the donor, aided by CD4^+^Th2 helper cells. This response leads to a cascade of events where B cells divide and differentiate into plasma cells, where a subset of activated B cells become memory B cells (63, 64). Preexisting or donor-specific antibodies (*de novo*) to donor human leukocyte antigen (HLA) lead to antibody-mediated rejection. Differentiated memory B cells and plasma cells produce *de novo* antibodies. In liver transplants, cellular toxicity is evident after an increase in the binding of donor-specific antibodies, and complement fixation occurs when expression of MHCII is upregulated after injury (65). Factors that can induce chronic antibody-mediated rejection are previous events of T cell-mediated rejection with insufficient immunosuppression and or developing chronic liver pathology (66). Recipients with antibodies to donor antigens bind to the endothelium of vessels of the graft in acute antibody-mediated rejection. This upshot is mediated by ABO-incompatible grafts or previously developed anti-HLA alloantibodies from previous pregnancy, transfusion, or transplantation cases.

### Immune memory in organ transplant

Immunological memory is a distinct characteristic whereby innate and adaptive immunity contributes to perseverance. Innate memory is classically known to generate a non-specific and rapid response. Previous encounters with pathogens develop adaptive features whereby the innate immune responses modulate upon re-infection, thus coining the term “trained” memory. This functional characteristic of “trained” memory is developed from the cells undergoing remodeling of chromatin structure and metabolic reprogramming. Hence allowing the innate immune cells to retaliate against re-infection with a stronger and more rapid response (67). Trained memory has been linked with acute rejection, whereas allograft survival is promoted upon suppression of trained immunity in organ transplantation.

Due to ischemic reperfusion injury, the donor organ releases DAMPs, which bind to PRP associated with trained macrophages, releasing pro-inflammatory cytokines in higher concentrations and activating adaptive immune responses. This results in allograft rejection (31). As this trained immunity will persist in the host for several months, the organ transplant might be subjected to long-term effects (68). The release of extracellular ATP during Ischaemic-reperfusion injury activates P2X7 (cell surface purinergic receptor for macrophages), producing cytokines. For example, IL-1beta and TNF contribute to allograft rejection (69, 70). Studies in mice have shown that blocking the receptor P2X7 demonstrated long-term transplant survival. Blocking P2X7 hinders T-helper1 and T-helper 17 T cell immune response, reducing the effecter T cell numbers and contributing to transplant survival (71). IL-6 demonstrated increased production by myeloid cells of mice, which, under oxidative stress, induces increased activation of allogenic T cells (72). Increased graft survival was observed in mice when IL-6 was strategically blocked post-transplantation. The blocking of IL-6 also leads to a decrease in the invasion of adaptive leukocytes (73).

Kidney transplantation surgery involves vascular tissue damage, induction, and infiltration of monocytes into the graft (74). Upon recognition of non-self alloantigens and DAMPs, these cells differentiate into various subsets of macrophages inside the transplanted kidney. The phenotype of these cells is also shown to be modulated by immunosuppressive agents in the recipient. If previously primed with donor cells, mice have been subject to stronger monocyte-mediated responses. Lymphocyte-deficient RAG (-/-) mice, when previously primed with allogeneic spleen cells or skin grafts, exhibited monocyte-mediated alloimmunity, i.e., allogeneic spleen cells were recognized and removed (75). As a result, these cells developed memory responses to counter the allo-antigens. Interestingly, it has further elucidated that the polymorphic variations in SIRPα (signal-regulatory protein-α) between donor and recipient monocytes in mice were identified by CD47 expressing monocytes of the recipient inducing innate immune responses against the allograft (76).

Upon contact with pathogen-derived ligands, the myeloid cells are subject to metabolic and epigenetic changes. These immunized cells produce a sturdier response to new infections and can reject allografts long term. Hence, functional reprogramming of the myeloid cells and macrophages over a long period contributes to their training, potentially playing a role in organ transplant rejection. Signaling cascades employing vimentin/HMGB1, oxidized low-density lipoprotein, and NOD2 (Nod-like receptor 2) have been implicated in the training of macrophages contributing to allograft rejection (30). The heart transplant model in mice has also shed light on the role of innate immune memory in rejection (31). Furthermore, the targeted mTOR inhibiting nanobiology of myeloid cells showed a reduction in H3K4me3 of genes associated with inflammation (e.g., TNF & IL-6) in the monocytes of the allograft. Here, the macrophages were induced with trained memory by vimentin (binds to dectin-1 receptor on endothelial cells) and HMGB1 (activates TLR4) that acted as mediators of inflammation in the transplanted heart (77, 78). This action reiterated the upregulation of vimentin and HMGB1 as inflammatory mediators, as reported in organ transplantation (79, 80). Treatment with vimentin and HMGB1 concerning organ transplantation can lead to the development of trained immunity (31). A crucial understanding was that organ transplant survival without the re-requisite of consistent use of immunosuppressive drugs could potentially be achieved when mTOR-inhibiting nanobiology specific to myeloid cells leads to an enhanced number of regulatory T cell population in the allograft (81).

Recently, scientists have shown that PIRA, an innate myeloid cell receptor, can recognize allo-MHC I molecules. Kidney and heart allograft rejection was reduced by deleting PIRA in the recipient or blocking donor MHCI binding to the donor RIPA receptor (82). Murine models have exhibited that allograft rejection is mediated upon organ transplantation, which induces the training of macrophages. Reports have indicated that trained immunity can be developed in hematopoietic stem, blood monocytes, and myeloid progenitor cells (81, 83). In parallel, more advanced adaptive immune memory is generated via epigenetic modifications and gene recombination, hence adding to its specificity.

In organ transplantation, trained immunity has been shown to modulate the fate of grafts, as demonstrated in animal models. More investigation into the intricate mechanisms of trained immunity at different regulatory levels in clinical settings is vital to identify potential targets for therapeutic interventions.

### Immune enhancement strategies in infection

It is well established that innate and adaptive immune responses are intricately interwoven systems aiming concordantly to improve survival. It is imperative to mention that the term “non-specific” in the context of innate immune response is under scrutiny as pattern recognition receptors (PRPs) expressed on the innate immune cells can specifically recognize the type of micro-organisms; for example, these cells can distinguish between gram-positive and gram-negative bacteria (84). Studies have demonstrated the adaptive property of innate immune responses, which signifies innate cells protect against infections (85, 86). In organ transplant, an infection after a kidney transplant can result in eventual organ rejection by the recipient owing to increased cytokine production by innate immune cells. Similarly, in the mice model, infection by *Staphylococcus aureus* simultaneously with skin transplantation led to increased IL-6 production and reduced graft acceptance (87). This outcome means macrophages-induced increased IL-6 responses combat *S. aureus* infection (88). Nevertheless, this high level of inflammatory cytokines can put into motion a mechanism inducing graft rejection in kidney transplants. Graft rejection in mice was seen as a result of immune response to *S. aureus* even with immunosuppressive agents (i.e., cyclosporine or sirolimus) support, indicating that these drugs could not regulate cytokine production by the macrophages. Following the above-mentioned studies, other research of murine models demonstrated graft rejection induced by costimulatory blockade resistance mediated by IL-6 produced by the macrophages (89).

A prospective strategy for immune enhancement can be achieved by exploring therapeutic targets in trained immunity that regulate innate immune responses. An intervention approach that can provide numerous possible targets is innate immune responses via cell surface molecules and soluble mediators that further modulate adaptive immune responses. In the case of infection, increasing the potency of immune responses in trained immunity can be targeted, whereas, in the case of organ transplantation, strategies targeting response inhibition leading to graft rejection can be evaluated. Some potential targets in the case of trained immunity include ligand-receptor interactions, epigenetic regulation, and metabolic wiring (81).

## Infections

### Infections before, at, and after transplantation and the infection’s timeline

In organ transplantation, infection prevention and management are critical for success. The risk of infection depends on the recipient’s immunosuppression regimen and local infection prevalence. Tailoring immunosuppression and advanced microbiologic diagnosis are crucial to addressing infectious disease challenges in transplant recipients (90).

Donor infections can be divided into “expected” and “unexpected” infections. The first category includes cytomegalovirus, HBV, HCV, *Toxoplasma gondii*, and BK polyomavirus. Present microbiological assays are available and can detect these pathogens. The expected infection category pathogens are reported to be of modest risk to the organ recipient and can be treated. In the second category, infections can occur due to the outbreak of known infections in new areas. This category includes viruses, bacteria, fungi, and parasites. Fewer than 1% of grafts are estimated to exhibit unexpected donor-derived infections. In cases where organs were sourced from a single donor, multiple recipients experienced a spectrum of infections, including M. tuberculosis, fungal pathogens, herpes simplex virus (HSV), human herpesvirus 8 (HHV-8), lymphocytic choriomeningitis virus (LCMV), rabies virus, Trypanosoma cruzi, microsporidiosis, human immunodeficiency virus (HIV), and hepatitis C virus (HCV) (91–97). Examples of unexpected infection include the spread of WNV in the USA and Chikungunya virus in Italy. At the time of the outbreak of these two infections, no diagnostic assays nor therapeutic option was available (98).

### Infected donor organ

Some donors have treatable infections, including bacteria (pneumonia or sepsis) and viruses (hepatitis B, HCV, or HIV). Recipients infected with HIV, HBV, or HCV and require antiviral therapies can be offered organs from donors infected with the same viruses. Currently, present treatments can control infection of syphilis or tuberculosis in recipients. The hallmarks of transplanting infected organs include delay in access to microbiological data at the time of transplantation or hampered testing, as seen in the case of HIV (2011).

Bacterial infections have been known to be controlled by applying surgical prophylaxis and antibacterial regimens during the transplantation period (99, 100). MRD includes spectrum β-lactamase (ESBL)-producing Enterobacteriaceae, carbapenem-resistant *Acinetobacter baumannii* (CRAB), *Klebsiella pneumoniae* (CRKP), and other carbapenem-resistant Enterobacteriaceae (CRE). The disease condition increases if an organ recipient is infected by MRD pathogen (101). Antimicrobials such as Colistin, tigecycline, and fosfomycin are presently the main agents potent against a relatively new highly resistant strain with a carbapenemase enzyme, i.e., NDM− 1 (New Delhi Metallo-β-lactamase− 1) discovered in 2008. In organ transplantation, NDM-1-related infection is yet to be recorded.

Donor organ-derived viral infections play a major role in modulating the immune responses in immune-compromised recipients. Higher frequency in the case of liver transplant recipients has been shown to acquire HBV when prophylaxis is not administered. Data demonstrate that HBV (*de novo*) was acquired in 58% of HBV non-immune organ recipients. However, only 18% of recipients who had been vaccinated acquired the infection, 14% of recipients were positive for HBV antibodies, and around 4% of recipients were naturally immune (102). Lamivudine (antiviral agent) is the favorable and cost-effective choice for antiviral therapy of anti-HBcAg-positive donors (103, 104). Donors infected with HBV, HCV, or HTLV (human T cell lymphotropic virus) require optimal prophylaxis (105–107).

### Risk of infection

Two main components encompass the risk of infection in the recipients after transplantation. One is the donor and recipient’s epidemiological exposure, including recent and remote exposures (92). Second is the overall immune suppression conditions and all related components that can add to the risks associated with infection. **Table 1** is an adaptation of previously reported work. It depicts the contributing agents in each component of the risk factors associated with organ transplantation (92) (90).

**Table 1:** Characteristics components reported as potential risk factors of infection in organ transplant recipients (some examples included).

Infection	Epidemiological exposure	Immunosuppressive net state
**Virus**	Herpes, i.e., Hepatitis viruses, Retroviruses, Others including West Nile (WNV), Chikungunya, Zika, Dengue, Community exposures Food- and water-borne, Respiratory viruses, Common viruses, Polyomavirus, papillomavirus	Immunosuppressive therapy constitutes the time, dosage, and trajectory of the spatiotemporal order of therapeutic agents. Immune disorders, including autoimmune disorders and immune system-related genetic polymorphism
**Bacteria**	Gram-positive and gram-negative bacteria, *Mycobacteria, Nocardia* species	Previous treatments, which include broad-spectrum antimicrobial drugs and chemotherapy-related drugs and procedure
	Methicillin-resistant staphylococci, Antimicrobial-resistant enterococci, Multidrug-resistant gram-negative bacilli*, Aspergillus* species*, Candida non-albicans* strains	Loss of skin integrity and breaks in mucosal barriers induced by catheters and drains
**Fungi**	*Candida* species, *Aspergillus* species, *Cryptococcus* species, Endemic fungi, Opportunistic molds, respiratory pathogens, Geographic fungi	Technical obstructions and drawbacks associated with surgical procedures lead to injury to graft, causing wounds and fluid collection. Immune disorders, including autoimmune disorders and immune system-related genetic polymorphism
**Parasites**	*Toxoplasma gondii*, *T. cruzi*, *Balamuthia* species, *Strongyloides stercoralis*	Metabolic dysfunction, including uremia and diabetes

### Infections- different types of microbiomes- donor and recipient

The microbiome collects microorganisms in the tissue and surfaces consisting of commensal flora and infectious agents. Infection from donor organs, latent infections (including fungal, viral, or parasitic), novel nosocomial or community-acquired infections, and previous colonization of mucosal surfaces are the primary sources constituting the microbiome of the organ transplant. It has been reported that this microbiome has a diverse role as it maintains a dynamic and regulated interaction with the immune system, which, upon shifting microbiome patterns, plays a key role in rejection in recipients of organ transplants by dysregulating the immune system. Immunosuppressive agents, exposure to infection, antimicrobial therapy, and surgery distort the normal microbiome of the recipient. The disrupted microbiome and induction of new immune system responses in this context alter the fate of the organ transplant (108).

### Donor- and recipient-derived infections

Microbiologic screening is vital as it provides information regarding the donor and recipient, enabling post-transplant preventive measures to be established (94, 109). Each strategy is personalized, for example, antifungal treatment in lung recipients and individualized antiviral prophylaxis for herpesviruses upon result analysis of pathogen-specific serologies of both donor and recipient (110, 111).

Donor infections can reappear decades after the first exposure, for example, in the cases of *Strongyloides stercoralis*, tuberculosis, or coccidioidomycosis. Donor colonized infections like *Aspergillus* in the donor’s lung can enhance graft rejection. Microbiologic assay and epidemiologic history are assessed to screen for common pathogens. Treatment strategies are implemented before the transplantation procedure in case of active infection. Perioperative prophylaxis is considered a strategy in the case of multidrug-resistant organisms, including MDRO or molds, which, under specific situations, can be used postoperatively, as seen in the case of *Aspergillus* in the recipient’s lung (100).

### Timeline of infection

After transplantation, the occurrence of infection as a function of time can be estimated when the recipients are on standard immune-suppressive regimens. Over time, the shifting balances between the risk factors [i.e., surgical procedure, hospitalization, immune-suppressive regimen, infections, and other factors (92)] provide predictive infection patterns. These are shifted via changes in the immune-suppressive regimen, infections of viruses, and epidemiological exposures. Antimicrobial prophylactic can serve to delay but not remove the infection. Upon suspension of prophylactic agents, the risk of infection decreases as the net state of immune suppression is reduced (112–114). The timeline of infection is represented in **Table 2** as a function of three overlapping periods for post-transplantation patients

**Table 2:** Timeline of Infection Phases and Associated Infections in Post-Transplantation Patients.

Phase	Post-Transplantation Period	Types of Infections	Key Information	References
**Phase 1**	30-day period post-transplantation	Surgical anomalies, donor-derived, nosocomial infections	Rare opportunistic infections, antibodies, fever, and graft rejection were observed	(90)
**Phase 2**	6–12 months post-transplantation	Various sources of infections, e.g., CMV, HSV, PCP, etc.	Differential evaluation of persisting infections; immunosuppression evaluation	(92, 115, 116, 117, 90)
**Phase 3**	6-12 months post-transplantation	Community-based exposures, CMV, recurrent infections	Recipients with optimal organ function have reduced risk; antimicrobial treatment	(1, 118)

## Fungal Infection

### Characteristics and evolution of fungal spectrum

Past decades have brought fungi into the limelight as human pathogens since over 1.6 billion annual deaths associated with fungal diseases implicate its critical role in human pathology (2017). In 2020, the World Health Organization (WHO) put forward a list of fungal priority pathogens, providing direction for future research into fungus-associated infections (119). As the number of immune-compromised patients has increased drastically, simultaneously fungi have emerged as infectious agents. It is imperative to decipher the virulence of fungi as human pathogens to understand the opportunistic actions of fungi.

With over 150,000 species and many yet to be discovered (120, 121), the kingdom of fungi presently consists of over 200 orders and 12 phyla (122, 123). Several hundred species are associated with human pathogenicity and death and are part of a few lineages. The fungal tree of life and plotting human pathogen-associated genera illustrated that the evolution in human pathogenicity has occurred in over 12 different lineages. Remarkably, human pathogenicity has evolved multiple times within a few of these lineages, indicative that these lineages possess distinct characteristics that prepare adaptation to human pathogenicity, for instance, *Aspergillus* fungi, whose pathogenicity has evolved independently many times (124). *Aspergillus fumigatus* and *Aspergillus flavus* are pathogens causing aspergillosis, whereas their immediate relatives are non-pathogenic (125–127). Pathogenicity has evolved independently within the clad of budding yeast around five times (128, 129), including Candidiasis *Candida (Nakaseomyces) glabrata*, *C. albicans*, and *C. auris* pathogens.

It has also been seen that within a particular lineage, several species exhibit human pathogenicity where close relatives, in many instances, demonstrate significant variations in the degree of virulence. Although the human pathogen *C. albicans* and *C. dubliniensis* are closely related, *C. albicans* has higher virulence (130). *Cryptococcus neoformans* is prevalent among inimmune compromised individuals, and *Cryptococcus gattii* infections among immune-competent individuals (131, 132). Furthermore, significant differences are seen in the pathogenicity and antifungal drug resistance spectrum within the 12 pathogenic species of *Aspergillus* section *Fumigati* (133). Virulence and drug resistance profiles of strains of *C. albicans* (134, 135) and pathogenic *Aspergillus* (136) demonstrate considerable heterogeneity in genome and phenotype. Hence, diversity in pathogenicity-related genes is present among species and lineages and between strains in the population of pathogenic fungi.

*C. albicans* and *C. dubliniensis*, being close relatives yet distinct in virulence, do not show significant variations in genetic content (137). Upon evaluating differences in the orthologous gene expression, it was evident that there was a high expression of 15 genes related to glycolysis in the case of *C. albicans*. In contrast, high virulence was achieved by engineering the high expression of 15 genes in C. dubliniensis (130). Changes in genetic traits can modulate pathogenicity and infection-associated features. Heterogeneity in the genome of *C. albicans* strains exhibited many genetic differences. For example, differences in virulence and infection-associated characteristics in strain were attributed to single nucleotide polymorphism (134)

### Common fungal infection

Approximately 5-42% of solid organ transplant recipients suffer from fungal infections, which is subjective to the type of organ transplanted and the state of immunosuppression of the recipient (138–140). Invasive Candidiasis (IC), Aspergillus species, Mucorales and Fusarium species, and *A. fumigatus* (Aspergillus) are the most common fungal infections in different organs of recipients. The occurrence of most common fungal infections in solid organ transplant recipients is represented in **Table 3**.

**Table 3:** Occurrence of Common Fungal Infections in Solid Organ Transplant Recipients.

Fungal Infection	Prevalence in Solid Organ Transplant Recipients	Percentage Range	Information	References
**Invasive Candidiasis (IC)**	Most common among intra-abdominal organ recipients	5% - 42%	*C. albicans* is prevalent, but non-albicans Candida species are emerging as causes of IC. *C. auris* outbreaks in intensive care units.	(138–140) (141–144 145–147, 148–150)
**Aspergillus species**	Prevalent in lung transplant recipients	Varies by organ type	Invasive pulmonary aspergillosis common in lung recipients. Increasing demand for antifungal susceptibility assays due to azole resistance.	(141–147, 151, 152, 153 154, 155, 156, 157, 141, 158)
**Mucorales and Fusarium species**	Associated with SOT infections	Not Available	Rare endemic pathogens like *Pneumocystis jirovecii* and Cryptococcus under investigation for their virulence in organ transplant recipients.	(153)
***A. fumigatus* (Aspergillus)**	Frequent in heart, lung, and liver transplants	5% (kidney) - 70% (heart)	Accounts for a significant percentage of IFIs in various organ transplants. Colonization rates vary by organ type.	(141–144)

### Timeline of fungal infection

Fungal infection cases have been reported within the first 90 days post-transplantation (159, 160). One month post-transplantation, invulnerability to Candida, Aspergillus, and Mucorales increased due to high levels of disruptions in barriers and changes in functional phagocytic activities (161). Disseminated aspergillosis infections in the central nervous system in recipients have also been described (162). During 1- 6 months post-transplantation, an increase in pathogenic infections is evident. Many infections are attributed to latent infections derived from endemic fungi and mycelial fungal infections (163, 164). The period post six months after transplantation is generally characterized by reduced fungal infection as recipients are under optimal immunosuppressive regimens. However, aspergillosis and opportunistic fungi infections are evident in 10-20% of recipients who experience neoplasia or are under high levels of immunosuppressive regimens (161, 163, 165).

Earlier reports related to *Aspergillus* have shown infection can occur in the first hundred days post-transplantation, e.g., 120 days in lung transplants, 82 days in kidney transplants, and 45 days in liver transplants. In subsequent years, reports have demonstrated *Aspergillus*’s bimodal trait of infection constituting an early onset and late onset infection. The early onset within the first month reflects potent environmental exposure (115). 51% of *Aspergillus* infections in lung transplant recipients are within the first 90 days post-transplantation, whereas 72% are within 180 post-transplantation (166). Early-onset infections are manifested as Tracheobronchitis or anastomotic. Invasive pulmonary and disseminated infections are seen later on.

### Risk factors leading to fungal infection in organ transplant

General risk factors associated with fungal infections in solid organ transplant recipients and their occurrence differ regarding two components. One is the recipient’s predisposition to infection, and the second is the degree of severity of exposure. The vulnerability of a recipient to fungal infection is governed by several contributors, mainly immune flaws caused by underlying disorders necessitating an organ transplant (167, 168), organ transplanted type, complications associated with surgery (152, 160, 167), metabolic changes, net state of immunosuppression, infections by viruses, loss of renal function and supplementary therapy to manage graft rejection (152, 165, 169).

Particularly, fungal infections in organ recipients are primarily influenced by two major risk factors: host-related and environmental factors. Host-related factors encompass various determinants, such as age, gender, genetic predisposition, underlying medical comorbidities, and the level of immunosuppression. For instance, individuals with compromised immune systems due to conditions like organ transplantation, HIV/AIDS, cancer, or diabetes exhibit an increased vulnerability to fungal infections. On the other hand, environmental factors encompass exposures to sources of fungal contamination within the surroundings, including soil, water, and air, as well as suboptimal hygiene and sanitation practices (170–172).

The *Aspergillus* species is known to colonize normal or immune-compromised individuals via inhalation of spores. The virulence varies among strains, and it has been shown that the degree of invasiveness is attributed to the elastase activity of *Aspergillus* strains (173). 95% of Invasive Aspergillosis cases indicate that the respiratory tract is the entry point (163). Tissue infection is followed by blood vessel invasion, leading to dissemination(158).

Preclinical risk factors associated with fungal infections are invariably intertwined with genetic elements that likely contribute to an individual’s susceptibility. For example, a distinct genetic variant within the TLR4 gene has been linked to an elevated susceptibility to invasive aspergillosis in individuals undergoing allogeneic hematopoietic stem cell transplantation (174). Furthermore, a separate investigation revealed a genetic polymorphism within the Dectin-1 gene correlated with an increased predisposition to invasive aspergillosis among patients afflicted with hematological malignancies (175). In clinical settings, healthcare-associated infections, primarily driven by Candida and Aspergillus, are common, especially in intensive care units, invasive procedures, and prolonged antibiotic usage. Organ transplant recipients are at risk due to immunosuppressive therapy. Broad-spectrum antibiotics disrupt microbial balance, increasing opportunistic fungal overgrowth risk, including *Clostridium difficile* infections. Invasive medical devices, like catheters and ventilators, can introduce fungal agents, often resulting in Candida bloodstream infections, supported by a study on general hospital patients’ risk factors, including antibiotics, catheters, glucocorticoids, immunosuppressive agents, and chemotherapy (171).

The vulnerability to infection is increased in solid organ recipients by macrophage dysfunction and neutrophils post-steroid usage. Disease progression of IA is credited to the function of T cell adaptive immunity marked by dysregulated production of Th (T-helper cell) cell cytokines (176, 177). In *Aspergillus*, Th1-related responses protect against *Aspergillus*, whereas disease advancement is mediated by Th2 responses (176, 178). Th1 responses are downregulated in SOT recipients administered calcineurin inhibitors and corticosteroids to avert graft rejection (179). Immunity against Aspergillosis is also conferred by TLRs (Toll-like receptors). Th1 cytokine responses are induced by TLR2 and TLR4 stimulated by *Aspergillus* conidia. The hyphae germination results in defected TLR4 signaling, consequently increasing the Th2 responses. In this context, hematopoietic cell transplant recipients from unrelated donors demonstrated a close correlation between donor TLR4 haplotype S4 and the risk of IA (174). The risk of IA is increased in SOT recipients with renal failure and hemodialysis where T cell replicative responses are diminished, leading to enhancement in activation-induced T cell death (180) (181, 182).

Hypoxia, ischemia, and microcirculation are critical factors influencing fungal infections in grafts, particularly in organ transplantation. These factors contribute to the local tissue environment and can significantly impact the ability of the graft to resist fungal infections (183–186). Understanding their roles is essential to manage fungal infection risks in transplanted organs effectively. In grafts, hypoxia can manifest due to compromised blood flow or vascular damage incurred during transplantation. This oxygen deprivation weakens the host’s immune response, hindering the effective migration of immune cells to the site of infection (187, 188). Fungal pathogens, such as Candida and Aspergillus, often possess adaptations that allow them to thrive under low oxygen conditions, capitalizing on the hypoxic environment within the graft to establish and propagate infections (188).

Ischemia, conversely, refers to a decrease in blood supply to a particular tissue or organ. It may result from vascular injury during graft surgery or the host’s immune system attacking the graft tissue. Grafts subjected to ischemia are more vulnerable to fungal infections due to their compromised blood flow, which restricts the delivery of oxygen and nutrients to the site of infection. Furthermore, the diminished oxygen levels in ischemic grafts weaken the host’s defense mechanisms, making it easier for fungal pathogens to colonize and thrive within the graft (184, 186). Microcirculation, comprising blood flow through the smallest blood vessels, including capillaries, plays a vital role in delivering oxygen and nutrients to tissues. When the microcirculatory system within the graft is damaged, it can result in tissue hypoxia and ischemia, creating an environment conducive to fungal infections. Fungal pathogens can exploit these conditions to infiltrate the impaired microcirculation and evade immune surveillance, leading to persistent and challenging-to-treat infections (189).

### Endemic, geographically restricted fungal infection

Certain dimorphic fungi are geographically restricted and, in the case of healthy individuals, cause pneumonia. In immune-compromised individuals (e.g., HIV, transplantation recipients, and corticosteroid treatment), these fungi cause progressive pulmonary and extrapulmonary diseases. These fungi include *Histoplasma, Coccidioides, Paracoccidioides, Talaromyces*, and *Blastomyces* species. Many factors, including race, ethnicity, and hormonal profile, impact the potential risk and intensity of infection, yet a complete understanding of the processes is lacking. Individuals of ancestries like African Americans, Native Americans, and Asians are at a higher risk of blastomycosis and disseminated coccidioidomycosis. Males are more at risk of developing chronic paracoccidioidomycosis (190). Immune responses against histoplasmosis, coccidioidomycosis, talaromycosis, and blastomycosis are governed by the coordinated interactions between fungicidal macrophages and T cells producing IFNγ. Predisposition to infection is mediated by gene mutations encoding IFNγR1, STAT1, STAT3, CD40L, or GATA2 (responsible for type 1 immune response), IFNγ neutralizing autoantibodies (191). Predisposition to high-intensity infection is also seen in applying TNF inhibitors and IFNγ-targeted biologic emapalumab (190). It has been shown that among approximately 50% of patients evaluated for Coccidioides, dissemination is attributed to dysregulation in TNF production owing to genetic variation in genes for β-glucan (192). Macrophage lead T helper 1 cell responses that facilitate metallothionein-mediated zinc sequestration, causing pathogen starvation to play a vital role in nutritional immunity to *Histoplasma*. Conversely, T helper 2 cell cytokine IL-4 encourages the pathogen to acquire zinc, promoting intracellular survival (193). *Blastomyces* employs several tricks to evade the immune responses, for example, GM-CSF inactivation, inhibition of CC chemokine recruitment by the monocytes, and the release of IFNγ mediated by CD4^+^ T cells blocked by BAD1 adhesin (194). A combination of IFNγ and dupilumab, i.e., IL-4/IL-3 receptor inhibitor, can promote remission in refractory disseminated coccidioidomycosis where inborn errors of immunity were detected in the child (195).

### Immune responses against fungal infection

Immune responses primarily begin when pathogenic fungi are recognized by host pattern recognition receptors (PRRs). These include Toll-like receptors (TLRs), C-type lectin receptors (CLRs), NOD-like receptors (NLRs), and retinoic acid-inducible gene-1 (RIG-1)- like receptors (RIRs). These receptors are present on innate immune cells such as monocytes, DCs, neutrophils, and macrophages in circulation or tissue-resident; upon recognizing fungal PAMPS, they lead to a specific immune response against the fungal infection. This initial line of defense is tailored mainly depending on the site of invasion and the type of innate immune cells recruited to the site (196).

The detection of fungi via ectodomain of TLRs signals the adaptor protein, i.e., myeloid differentiation primary response 88 (MyD88), triggering a signaling cascade that activates the NF-K beta and MAPKs, leading to a response against the fungi. MyD88 knockout mice are prone to multiple fungal infections, demonstrating the vital role of MyD88 in initial responses (197, 198). However, it has also been shown that activation of NF-Kβ and MAPKs can be carried out via an alternate route employing TRAF 6 and TRAF3 to combat fungal infection (199). Sensing and elicitation of antifungal response are also carried out by CLRs consisting of Dectin1,2, macrophage-inducible C-type lectin (Mincle), and dendritic cell-specific intercellular adhesion molecule-3-grabbing nonintegrin (DC-SIGN). The carbohydrate component of the fungi is sensed by the C-type lectin-like domain (CTLD), where the CLRs activate Card9/Bcl10/Malt1 (CBM) signalosome followed by the NF- pathway either via Dectin 1 or Dectin 2 and Mincle (200). It has been shown that this CBM plays an important role in protecting against fungi in mammalian cells. Vulnerability to fungal infection has been seen in cases where loss of function of Card 9 is reported (201). When PAMPs are recognized by NOD1 and NOD 2 receptors, the Nodosome signaling complex activates the NFKβ and MAPK pathways (202). A Multi-protein complex called inflammasome that consists of NLRPs, apoptosis-associated speck-like protein (ASC), and inflammatory protease caspase-1 can distinguish the invasive fungi by identifying the hyphae of fungi responsible for the invasion. NLRP3 inflammasome is widely studied in this context and is known as a prototypic inflammasome. Once the NLRP3 inflammasome is activated via a plethora of upstream events, procaspase-1 activates the active caspase-1, promoting the maturation and release of IL-1β and IL-18 (203). A. *fumigatus*, C. *albicans*, and C.*neoformans* infections were seen in mice knockout of NLPR3, ASC and caspase-1, hence signifying the role of inflammasome in countering fungal invasion (204–206). It is imperative to mention that effective fungal removal is tackled by the cumulative overlapping interactions of the PRRs as the first line of defense; for example, in the case of *Histoplasma capsulatum*, a response is initiated via Dectin 1 in collusion with complement receptor 3 (CR3) activate the Syk-JNK-AP-1 signaling cascade and lead to tumor necrosis factor (TNF) alpha and IL-6 release (207).

Fungi components, once recognized, lead to the activation of oxidative and non-oxidative processes in innate immune cells that promote the removal of fungi. One strategy is phagocytosis by cells such as macrophages and neutrophils, whereby fungi, captured by phagosomes, are subject to lysosomal vesicles that induce fungal clearance (208). Macrophages and neutrophils also induce ROS production via the nicotinamide adenine dinucleotide phosphate (NADPH) oxidase complex, which kills the fungi by protein cross-linking and fragmentation (209). NADPH oxidase loss, either complete or partial, has been associated with *aspergillus* infection (210). Aside from the oxidative mechanisms, innate immune cells employ non-oxidative processes to kill invading fungi. Cathelicidin LL-37, histatins (Hst), and defensins are antimicrobial peptides (AMPs) that can kill pathogenic fungi. C. *albicans* undergoes loss of nucleotides and proteins when LL-37 attaches and breaks down the membrane (den Hertog et al., 2005), whereas Hst5 is taken up by the C. *albicans* and leads to fungi death by the production of ROS intracellularly and ATP efflux from the mitochondria (211).

DCs, as antigen-presenting cells (APCs), serve to present fungal components to the adaptive immune cells and promote long-lived memory, a protective mechanism against re-infection. In the case of *Candida*, the DCs take in the invading yeast by phagocytosis, promoting the induction of cytokines IL-12, which leads to protection by T helper 1 (Th1). If the Candida hyphae are taken up by zipper-type processes (including FcγR and CR3), IL-4 and IL-10 are generated, resulting in Th2 and Treg responses (212). The release of type I interferon (IFNα) and TNFα is promoted by plasmacytoid DCs, which protects against A. *fumigatus* in mice (213). *Blastomyces dermatitidis* antigens enter the lymph node via monocyte-derived inflammatory DCs where CD4^+^ T cells are primed by lymph node-based DCs (214).

IFNγ and TNFα release from immune cells play a vital role in removing fungal pathogens. Upon infection of *Paracoccidioides brasiliensis* and *H. capsulatum*, IFNγ enhances the expression of MHCI, promoting antigen presentation, phagocytosis, and macrophage responses (215–218). *H. capsulatum* and *aspergillus* infection are hindered by TNFα which simultaneously reduces regulatory T cell effects and enhances the recruitment of immune cells and the production of ROS (178, 219, 220).

The production of IL-17 by Th17 cells contributes to protection against infections by mucocutaneous fungi. Oropharyngeal candidiasis in IL-17A receptor knockout and Th-17 deficient mice demonstrated suppressed responses of neutrophils and diminished survival (221). On the other hand, *C. albicans and A. fumigatus* vulnerability is enhanced, where Th-17 has been shown to disrupt Th-1 responses (222). Hence, the exact role of Th-17 against fungi is yet to be explored.

CD8^+^T cells enhance immune responses against *pneumocystis carinii* (223), whereas these cells release IFNγ that promotes killing C. *neoformans* (224). Although several studies have demonstrated the antifungal role of antibodies, the exact role is still unclear. Ig M antibodies against carbohydrate components of the fungal wall lead to an increase in APCs movement to the lymph nodes where differentiation of Th-2 and Th-17 is promoted, as seen in infections associated with P. *murina* (225). Protection was observed in rats with vaginal candidiasis, where adoptive transfer of B cell therapy was carried out (226). C. *albicans* binding to oral epithelial cells has been blocked by IgA derived from human milk (227). Further research in adaptive immune responses against pathogenic fungi is anticipated to shed light on the precise role of different components of immunity.

The development of memory T-cells is integral to establishing antifungal immunity, a pivotal defense mechanism against fungal infections, particularly within immunocompromised individuals (228). Memory T-cells, a specialized subset of T-lymphocytes, can recognize specific fungal antigens through their T-cell receptors (TCRs) upon fungal intrusion. Initial exposure to these fungal antigens typically occurs during the primary infection or immunization event (229). Upon the first encounter with a fungal pathogen or following fungal vaccination, naïve T-cells undergo activation with specificity toward the pathogen in question. This activation process often necessitates the involvement of APCs, such as DCs and macrophages, which present the fungal antigens to the naïve T-cells, thereby prompting their activation (228, 230).

During the primary infection or immunization, a fraction of the activated T-cells undergo differentiation into memory T-cells. These memory T-cells are characterized by their long-lived presence, enduring within the body over an extended duration, often for a lifetime. Two principal categories of memory T-cells exist: central memory T-cells (T_CM) and effector memory T-cells (T_EM) (231–233). T_CM cells reside primarily within lymphoid tissues, such as lymph nodes and the spleen. They function as a reservoir of antigen-specific T-cells, facilitating their rapid expansion upon re-exposure to the fungal pathogen. In contrast, T_EM cells inhabit peripheral tissues, including those most susceptible to fungal infections. These cells are poised for immediate response to the pathogen and can execute effector functions, including the release of cytokines and cytotoxic activities, without the requirement for further differentiation (231–233).

After organ transplantation, if the transplant recipient encounters the same fungal pathogen, the memory T-cells tailored to that pathogen undergo prompt activation. This activation culminates in a notably accelerated and robust immune response compared to the initial infection (30, 234). Memory T-cells are instrumental in the secretion of cytokines, such as interferon-gamma (IFN-γ) and interleukin-17 (IL-17), which aid in recruiting and stimulating other immune cells to counter the fungal infection. In instances where the immune response, encompassing memory T-cell reactivity, proves inadequate for controlling a fungal infection, antifungal medications may be warranted. These pharmacological interventions are efficacious in containing the fungal infection during immune recovery (228, 229, 235).

### Key immunomodulatory approaches adapted by fungal infection

**Figure 2:**
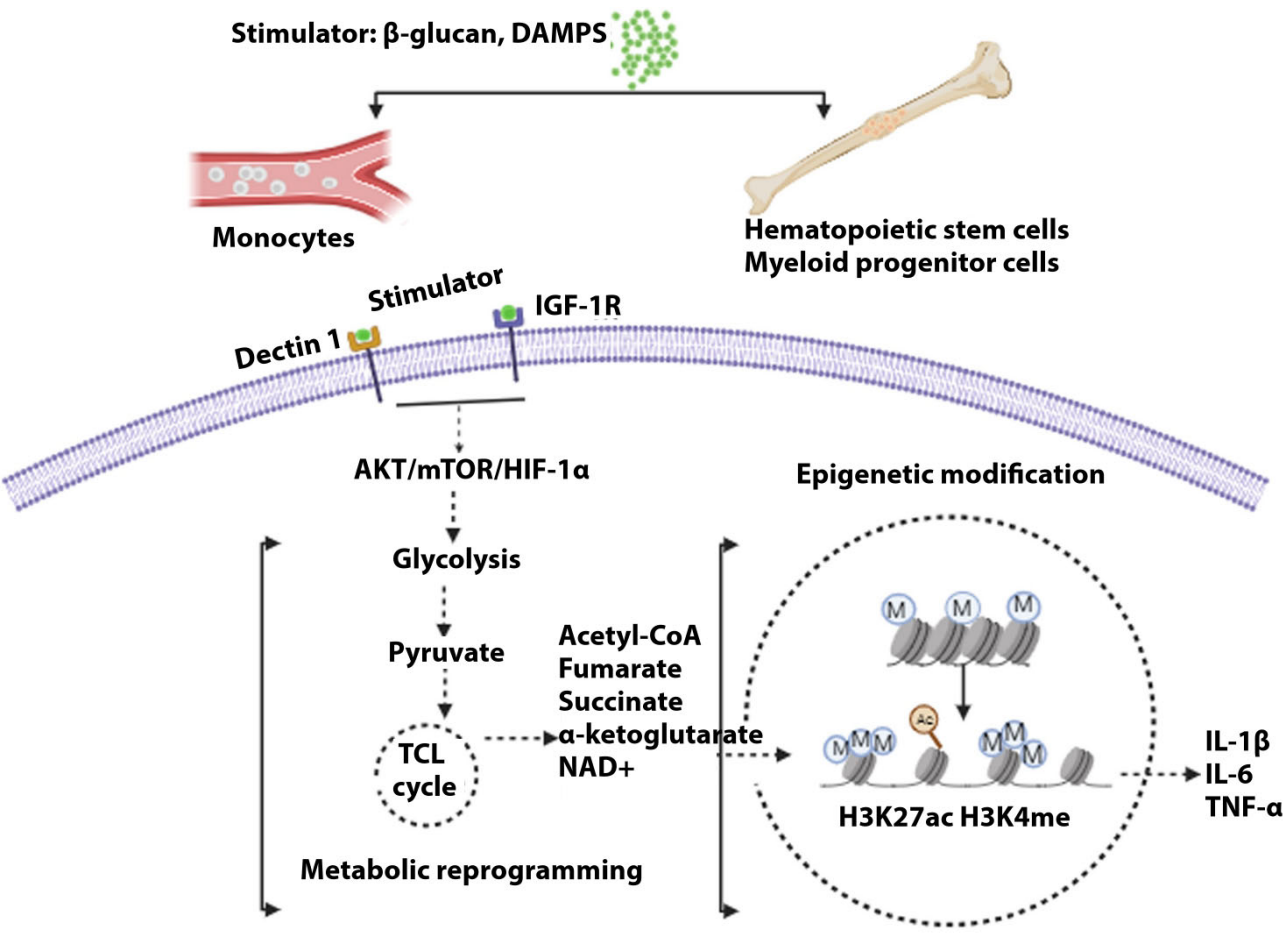
General mechanism of trained immunity. Stimulations of different cells, i.e., monocytes in blood or hematopoietic cells and myeloid progenitor cells (central), lead to the development of trained immunity. Upon induction of receptor signal by myeloid cells, signally pathway AKT/mTOR/HIFα leads to glycolysis. Pyruvate, a metabolic intermediate, enters the tricarboxylic acid cycle, resulting in several metabolites that modulate histone acetylation and methylation, resulting in the activation of genes governing inflammatory cytokines.

Fungi employ distinct mechanisms that cumulatively enable them to establish an infection. It is imperative to understand the pathophysiology that drives infections. The pathogenic potential of fungi is instigated by the main factors: immune system evasion, host target modulation, and host exploitation to access nutrients (**Figure 2**). The first point of contact of fungi is its cell wall, which forms contact with the host cell. Depending upon the type of pathogen, the cell wall components can constitute chitin, glucans, polysaccharides (e.g., mannoproteins), pigments, and waxes (236). Pathogenic *Candida* species camouflage their immune-stimulatory component of the cell wall, which is β-glucan, with mannoproteins—a strategy employed to prevent binding of β-glucan by PRRs, i.e., dectin-1 in humans (236). *Aspergillus fumigatus*, the β-glucan layer, is covered in hydrophobin RodA (237). A polysaccharide-based capsule is used to mask the β-glucan in the cell wall by *Cryptococcus neoformans* (238). Coating of α-1,3-linked glucans to shield the β-glucan, thus inhibiting the immunostimulatory cues, is seen in *Histoplasma capsulatum* (239). Furthermore, *Histoplasma capsulatum* diminishes the exposed β-glucan surface by Eng1, a β-glucanase (240).

Evasion of the host immune system plays a defining role in modulating the overall immune responses. *C. albicans* employ strategies to tackle the complement system by secretion of several proteins. *C. albicans* inhibit the activation of the complement system, opsonization, and phagocytosis of fungus by secretion of Gpm1 and Pra1 that bind to FH (host complement factor) and other complement factors (241). The Aspf2 factor engages FH in *A.fumigatus* (242). Dissemination of *C. neoformans* is promoted when anti-phagocytic effector protein App1 binds to macrophage receptor CR3 (complement receptor 3), preventing uptake and leading to early infection (243). In *A. fumigatus*, acidification in phagolysosome is repressed when DHN melanin hampers the endocytic pathway after the uptake by the immune cells (244). The activation of LC3- LC3-associated phagocytosis is hindered by DHN melanin in the case of *A.fumigatus* where phagocytosis plays an important role in defense against the fungi (245).

Pre-emptive measures to inhibit an immune response are fruitful; however, in cases where an immune response is activated, fungi can counterattack by either hindering the signaling mechanism or targeting proteins involved in host defense. In human pathogenic fungi, detoxification of the oxidative killing mechanism is done. In *C. albicans*, this detoxification is achieved by Sod 4 and 5 (surface-bound superoxide dismutases) (246, 247) or by Grx2 (glutathione reductase) and Trx1 (thioredoxin) (248). Sod 1 in *C. neoformans* (249), Sod1 and Yap1 in *C. glabrata* (250), and Sod 3 in *H. capsulatum* (251) demonstrate similar detoxification strategies. A secondary metabolite gliotoxin in *A.fumigatus* hampers the ROS production by the neutrophils (252). In *A.fumigatus*, DHN melanin hinders the activation of apoptosis by the macrophages by the PI3K/Akt cascade (253), diminishing the presentation of pathogen-derived antigens to the DCs. This action hampers the innate responses linked to the adaptive immune responses (254).

Upon infecting the macrophages, the fungi induce Pyroptosis (the programmed pro-inflammatory host cell death). *C. albicans* has shown documented evidence of pyroptosis. Fungal cell wall composition and hyphae formation play a role in triggering pyroptosis (O’Meara et al., 2018, 255, 256). This innate immune cell death is induced by several cues provided by the fungi, enabling the fungi to escape the volatile environment inside the immune cells. Upon infection by *C. albicans*, Saps (secreted aspartyl proteases) activate NLRP3 inflammasome, inducing pyroptosis (257). The amino acid transport transcriptional regulators Ahr1 and Stp2 contribute to activating inflammasome-inducing pyroptosis by hampering phagosomal acidification (258). However, upon pyroptosis, certain pro-inflammatory cytokines are secreted, leading to the recruitment of neutrophils and pathogen elimination.

Pathogenic fungi can reside and replicate inside immune cells for a long time without activation of host cell death programs, which is triggered eventually owing to massive host cell damage. *C. neoformans* can live and proliferate inside the acidic phagolysosomes via an optimal pH set by a fungal capsule buffer system. Thus, the fungi use the host as a safe replicative niche (259). *C. glabrata* residing in non-acidified phagolysosomes exploit macrophage cytokine patterns, leading to a less pro-inflammatory profile. Fungi residing and replicating inside the phagocyte lead to the lysis of host cells, and pathogens are released 2-3 days post-infection (260). Fungal biotin homeostasis in the case of *C. glabrata* has been demonstrated to evade the host’s immune response (261). Interestingly, C. albicans form hyphae upon phagocytosis, which mechanically damage the membrane within hours of infection (262).

It is important to note that pathogenic fungi can modulate the host cell fate, where in later stages of infection, phagocytosis is favored, and *C. neoformans* employ macrophages as Trojan horses to go through the blood-brain barrier (263). This pathogen utilizes vomocytosis (non-lytic expulsion) to escape from the phagolysosome in 10-27% of cases and within the initial 10 hours of infection (time varies between host types) (264). Vomocytosis has been reported in immune cells infected by *C. albicans*, *C. krusei*, and *C. parapsilosis*; however, the modulating factor involved has yet to be elucidated (265–267). Later studies showed that the peptide toxin candidalysin produced by C. albicans disrupts the phagocyte membrane, promoting fungal escape independent of PCD cascade activation (268). Human fungal pathogens in principle, modulate the immune responses upon activation and employ strategies to evade recognition, break free from immune cells and disseminate into the host.

The dissemination process is attributed to certain fungal components that modulate the fungal uptake in the host. An active invasion of host tissue by the growing hypha utilizes the increasing force of the hypha for active penetration. This penetration is seen in the case of *C. albicans*, where invasion is facilitated by the transition of yeast to a hyphal architecture (269). *C. albicans*’ ability to employ active invasion and induced endocytosis is scarce among fungal human pathogens (270). Another modulatory mechanism for dissemination is the use of urease by *C. neoformans*; the endothelial barrier is compromised by fluctuating ZO-1 protein balance that invades the brain tissue of the host (271).

Toxins are important in escaping and inducing immune responses, damaging the host, and acquiring nutrients from the host. Candidalysin plays a governing role in host cell damage and mucosal infections (272, 273). Danger response pathways are activated upon the immune responses induced by the destruction caused by candidalysin (272). This danger response signifies the role of candidalysin as virulent and nonvirulent (274). An important feature of pathogenicity is pickpocketing of metals, for example, iron, by a scavenging system constituting of proteins, hemophores (heme extraction) by Rbt5, Pga7, and Csa2, and Cfl1 and Cfl95 (ferric reductases) and the ability to trap ferricrocin (xenosiderophores), for instance. This feature is elaborately studied in *C. albicans* (275, 276).

During a fungal infection, the cell wall composition adapts to the variations in the environment, hence constituting prey for the immune system. β-glucan in fungal cell walls has been reported to mediate trained immunity that leads to the reprogramming of innate immune cells, which, upon secondary encounter with a pathogen, results in an adaptive response (277). Inflammasome activation disruption is seen by β-glucan mediated trained immunity (278). β-glucan, as reported earlier, triggers NLRP3 inflammasome (279), and the inflammasome is inhibited via trained immunity (278). Therefore, it is imperative to understand the overall effect of β-glucan modulating the activation of inflammasome and counteract through inducing trained immunity responses. The discovery of a distinct mannan oligosaccharide in *C. auris* was shown to play a significant role in generating stimulus for the release of cytokines (280). The role of the two components of the cell wall, i.e., mannan and β-glucan, in modulating the immune cell responses requires further investigation. In *A. fumigatus*, melanin is a component of the conidia and also in the cell wall of pigmented fungi (281). Recent studies have shown that this melanin is crucial to inducing macrophage metabolic reprogramming that results in enhanced glycolysis upon induction of host defense (282). It was also shown that irrespective of the accessibility of C-type lectin receptor MelLeC, melanin orchestrated the intracellular calcium, leading to hypoxia and activation of the mTOR cascade. Hence, evaluating the cumulative role of the fungal cell wall components in immunomodulation tactics is critical.

T cell immunometabolism plays a crucial role in various infections in transplantation, with recent studies suggesting that changes in intracellular metabolic programs control T cell activation, proliferation, and differentiation into T effector (Teffs) or T regulatory cells (Tregs) (283–285). The metabolic differences between Tregs and Teffs can influence the balance between immune tolerance and rejection in organ transplantation (286; KAZMI et al., 2020). While this is well-established in the context of graft rejection, there is limited research discussing the specific modulation of T cell metabolism during the ischemic phase of IFI in transplantation. However, during IFI in transplantation, T cell metabolism is influenced by several key factors during the ischemic phase (287). First, ischemia induces tissue hypoxia, triggering the activation of hypoxia-inducible factors (HIFs). This activation drives T cells to shift toward glycolysis, providing the energy required for immune responses against IFI (288). Additionally, immunosuppressive medications, administered to prevent graft rejection, can indirectly disrupt T cell metabolism, potentially compromising their ability to combat fungal pathogens (289). Moreover, the ischemic phase can result in nutrient scarcity within the graft and surrounding tissues, affecting T-cell functionality (290). Lastly, fungal pathogens such as Candida and Aspergillus exploit host immune cells’ reliance on glycolysis in hypoxic conditions, evading immune clearance (291). Understanding these intricate interactions is crucial for enhancing our approach to fungal infections following transplantation.

### Current models of diagnosis, treatment, and follow-up

Fungal pathogens are naturally commensal to the human body. Some are prevalent in the environment, making early detection more challenging. Ideally, the diagnosis should include detecting stages where fungi become pathogenic and develop invasive potential. Understanding fungal infection biology through sequencing technology, metabolomics, and advanced imaging techniques is vital. In parallel, the immune responses in fungal infection are crucial to elucidate the mechanisms that can be explored for therapeutic potency by developing advanced diagnostic tools and specific drug targets. The ability of the innate responses to shield adaptive immune-deprived individuals can be investigated by devising specific sensors (i.e., fungus-specific CD4 T cells) as fungal infection diagnostic tools.

Currently, clinicians divide patients into at-risk groups to ensure targeted plans for diagnosis, prophylactic treatment, and pre-emptive therapies. The diagnosis technology has been upgraded where valuable histopathology of infected tissue can be retrieved for deep insight (e.g., robust and validated culture techniques, PCR matrix-assisted laser desorption/ionization time-of-flight mass spectrometry (MALDI–TOF MS), T2 MRI technology and genome sequencing). Pre-emptive therapy is critical where promising biomarkers, such as β−d−glucan, galactomannan, and mannan as agents, can control infection early on. The invasive character of fungi can thus be blocked by controlling infection (292).

Fungal-infected patients are treated with antifungal agents (i.e., polyenes, azoles, flucytosine, and echinocandins) as the first line of therapeutic agents. However, they provide limited scope due to toxicity, emergence of resistant strains, and other factors. Immuno-modulating drugs are being investigated as an adjunctive treatment to typical antifungal agents. It is postulated that using two prolonged therapies can, to a greater degree, lift the immune responses in patients, especially immune-compromised individuals. It is, however, imperative to mention that as the antifungal agents provide a certain degree of potency, the use of immuno-modulating drugs can be clinically demanding. Cytokine-based therapy is the induction of cytokines, which promotes proliferation, differentiation, and activation of immune cells to retrieve or amplify immune responses to fungal infection. These include CSFs (Colony-Stimulating factors), IFNγ, TNFα and IL-12. Improved clinical outcome was seen in the case where G-CSFGranulocyte–macrophage CSF) in combination with fluconazole or amphotericin B was administered for refractory mucormycosis in leukemia and neutropenic patients (293). Short-term induction of IFNγ in conjunction with amphotericin B prompted the removal of *C. neoformans* from HIV patients (294). TNFα can be explored for clinical application as administration of TNFα in wild-type and neutropenic mice showed protection against infection of *A. fumigatus* (295). IL-12, in conjunction with fluconazole, demonstrated protection against invasive candidiasis in neutropenic mice (296). However, its clinical role remains a challenge as IL-12 can promote IL-10 (anti-inflammatory cytokine), IL-12 can potentially instigate predisposition of individuals to infection (297).

**Figure 3:**
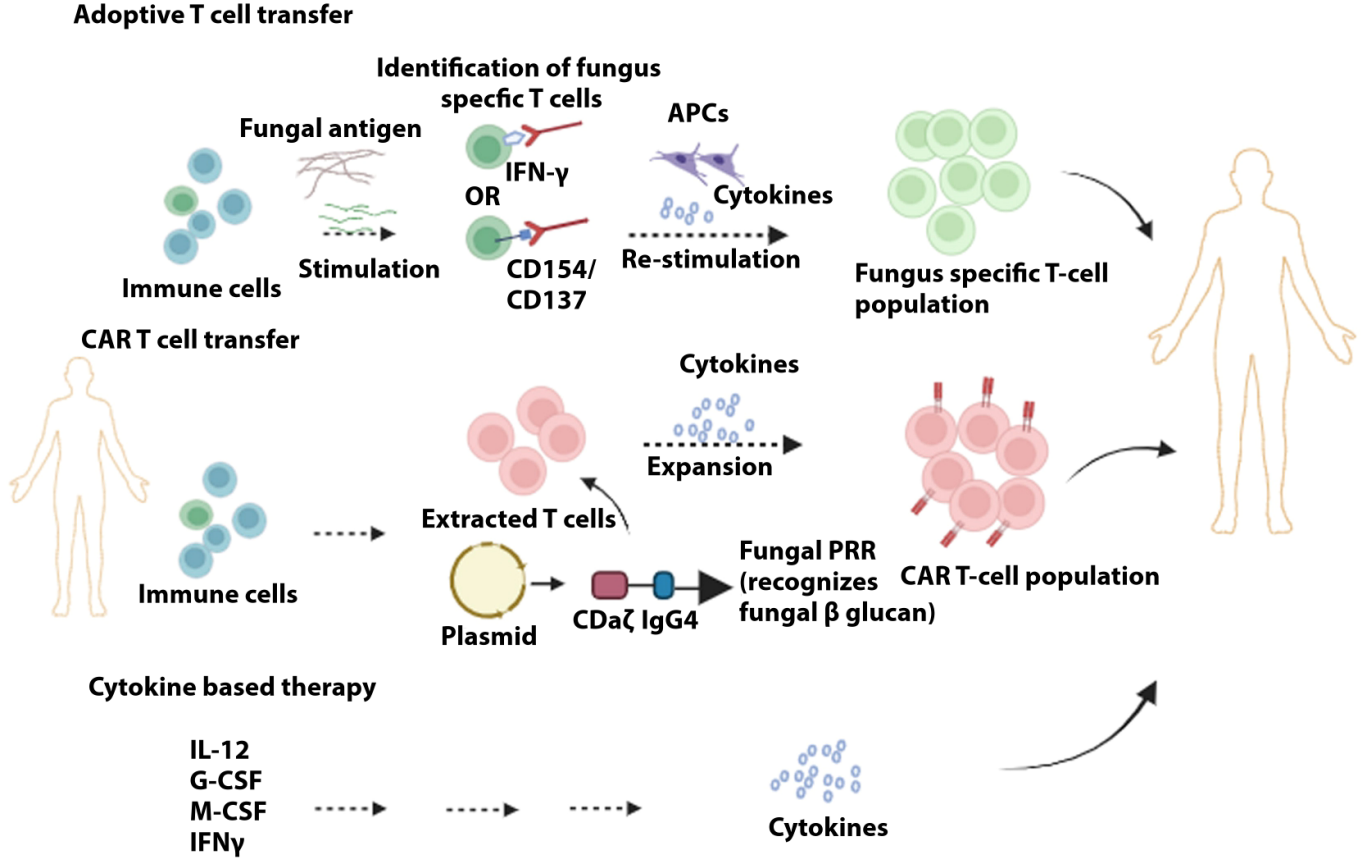
Immunotherapies to target and prevent fungal infection. In adoptive T cell transfer, initial antigen-specific T cells are stimulated with fungal extracts. Sorting of stimulated T cells via specific markers (e.g., CD154/CD137) is followed by further stimulation and clonal expansion of the fungus-specific T cell population. Chimeric antigen receptors are produced containing the domains identified by target antigens (e.g., dectin-1 recognizes fungal β-glucans), whereby upon attachment of these receptors to T cells, these fungus-specific antibodies are expanded. Administration of cytokines can provide the optimal repertoire of immune cells to counter fungal infection

Cell-based therapy includes adoptive T-cell therapy, Granulocyte transfusion, CAR-Chimeric antigen receptor T-cell therapy, and mAbs (monoclonal antibodies) (**Figure 3**). Adoptive T cell therapy poses challenges in clinical settings as the large-scale expansion of low-tier fungi-specific T cells is strenuous. In this treatment, GvHD (graft versus host disease) can be instigated, and the anti-GvHD prophylaxis might hamper the role of infused T cells (298). In preclinical studies in immune-deficient mice, the D- CAR^+^ (CLR Dectin-1-specific) T cells, upon activation by β−glucan, release IFNγ and diminish *A. fumigatus* growth (299). CAR-T cell therapy has tremendous potential as a therapeutic agent but is associated with cytokine release syndrome and neurologic toxicity that constitute dire side effects (300). Added limitations that need to be addressed include the extended timeline for developing autologous CAR-T cell repertoire and the high cost of CAR-T therapy. Risk factors associated with an increase in invasive fungal infection include dysfunction of neutrophils and neutropenia, where the disease progression is reduced when granulocyte transfusion can be applied as adjunctive treatment. This treatment has been demonstrated in children suffering from neutropenia, effectively treated with granulocyte transfusion combated invasion infection (301). Increased protection against invasive fungal infection was also seen when this treatment was administered to stem cell patients (302). Presently Mycograb (i.e., Efungumab) and 18B7 are the only two mAbs that have paved their way into clinical trials. The lack of elaborate understanding of humoral immunity against fungal infection, in parallel with the high cost of production, has proven to be the main hindrance to effective advancement in this field. Efungumab targets the heat shock protein 90. The human recombinant antibody is potent against *Candida* species, including *C. albicans, C. krusei, C. tropicalis, and C. parapsilosis*. In an interesting study, it was demonstrated that the use of Mycograb in conjunction with amphotericin B enhanced the clinical outcomes by 84%. It was only 48% with amphotericin B for patients with invasive candidiasis. The death rate was also reduced considerably (303). However, due to inconsistencies in quality control, Mycograb has yet to reach the market (304). 18B7, a murine antibody, targets the polysaccharide capsule of *C. neoformans* (305). Clinical trials of HIV patients with cryptococcal meningitis were shown to be tolerant of 18B7, and high doses led to a reduction in serum cryptococcal antigen (306). 18B7 has yet to reach the clinical setting owing to a lack of developmental support for the antibodies (307).

Although the burden of high-risk populations is increasing, no clinically approved vaccine has reached the market in case of protection against potential invasive fungal infection (308). Vulvovaginal candidiasis affects around 50-70% of women at least once in a lifetime (226, 309). PEV7 is a vaccine composed of truncated Sap2 recombinant protein set up on virosomes that can produce specific antibodies IgG and Ig A that confer protection against vaginal candidiasis. In phase one of clinical trials on rats, PEV7 demonstrated favorable efficacy and encouraging results (310). NDV-3 vaccine constitutes *C. albicans* Als3p (agglutinin-like sequence 3 protein) initially reported to exhibit protection against *C. albicans* infection in mice. Phase 1 clinical trial showed that 40 healthy individuals favorably tolerated NDV-3. Compared to the placebo group, enhanced levels of IgG and IgA and increased levels of IFNγ and IL-17A by T cells were observed (311). These results supported the organization of phase 1b/2a clinical trials for NDV-3. Women with recurrent vaginal candidiasis were treated with NDV-3, and the vaccine proved efficient and safe (312). PEV7 and NDV-3 vaccines are licensed to NovaDigm Therapeutics Inc., and the target is to introduce a multivalent fungal vaccine conferring protection against *C. albicans* (310).

Recombinant antigen vaccine made up of Ag2/PRA (antigen 2/PRA) and CSA (Coccidioides-specific antigen) has shown favorable survival in mice suffering from coccidioidomycosis (313), caused by the endemic fungi *Coccidioides immitis* in immune-competent individuals in the southwestern United States and northwestern Mexico. In 2006, a patent was granted for Ag2/PRA1-106 as a vaccine antigen candidate (307).

Pan fungal vaccines that can potentially target several clinically relevant fungi are yet to reach clinical trials. Derived from the KEX1 sequence that is conserved in numerous pathogenic fungi, NXT-2, a recombinant peptide vaccine, has exhibited increased protection against invasive aspergillosis, systemic candidiasis, and Pneumocystosis in murine and nonhuman primates (314). Pan fungal vaccine based on β-glucan (i.e., CRM197) has demonstrated efficacious protection against *C. albicans* and *A. fumigatus* in mice (315, 316). As β-glucan is known to induce trained immunity, pan vaccines based on β-glucan can potentially protect against other infectious agents, such as *Mycobacterium tuberculosis* (277).

## Strategies enhancing immune response, management, and addressing challenges related to fungal infection in organ transplant

### Mechanisms for detection of fungal infection

Early diagnosis is crucial for developing therapeutic strategies for immune-compromised organ transplant recipients. Generally, detecting fungal infections involves evaluating individuals at risk, microbiological evidence, and suspicious assessments in a clinical setting. Radiographic imaging is also required in cases where the possibility of deep penetration of pathogen is to be assessed, for example, in cases of lungs (317). The gold standard for diagnosis is direct detection (i.e., histology of infected tissue or culture from a sterile place). However, its unavailability or lack of sensitivity poses limitations. Cultural growth assay from nonsterile sites also poses limitations as it is difficult to elucidate between infection that is either colonized or an invasive infection. Testing is possible for fungal antigens using 1,3-β-D-glucan from serum, galactomannan from serum or BAL, or antigen detection by lateral flow device assays from BAL specifically for *Aspergillus* (318, 319). Conclusive interpretation is only possible when data from mycological tests is considered cumulatively with evidence from clinical and radiological evaluations.

*Candida* pathogens are most commonly (*C. albicans, C. glabrata, C. tropicalis, and C. krusei*) determined by a T2 Candida assay (T2 Biosystems). An FDA-approved test has shown 89-91% and 99% sensitivity and selectivity in clinical trials upon assessing whole blood samples (320, 321). Owing to the NPV (strong negative predictive value), this test is beneficial in withholding or discontinuing treatment decisions. *C auris*’s precise detection via TaqMan real‐time PCR assay is an important diagnostic tool (322). Similarly, specific TaqMan Real-Time PCR can detect very low tier of *A. fumigatus* (323). LED (lateral-flow device) is a recent reliable diagnostic tool in the case of IA (324) (325), detecting glycoproteins antigen in serum and BAL of patients. The cost-effectiveness, ease of use, and short detection time make this technique promising (326). Additionally, in consortium with quantitative PCR, invasive pulmonary aspergillosis can be detected (327). The drawback of LED is that when antifungal is administered, LED sensitivity is hampered (328).

### Immune responses and immunotherapeutic interventions

When an invasive fungal infection is suspected based on the relevant diagnostic tools, it is critical to initiate an early pre-emptive therapy to avoid worsening clinical outcomes.

For Candida infections, three classes of antifungal agents are present. These are azoles (fluconazole, voriconazole, posaconazole and isavuconazole); echinocandins (anidulafungin, caspofungin and micafungin); and polyenes (amphotericin B deoxycholate and lipid formulations of amphotericin B). Developing an empirical therapy for SOT patients with either suspension or confirmation of an IC infection is imperative to identify the prior exposure of recipients to antifungal agents within the past 90 days and to avoid resistance to the designed therapy (329, 330). The results of randomized clinical trials comparing amphotericin B, fluconazole, and isavuconazole (331–333) led the Infectious Diseases Society of America to put forward treatment guidelines for candidemia and other types of IC (334). According to these recommendations, Echinocandins should be administered as the first therapeutic agent. Micafungin and caspofungin have shown similar clinical safety and efficacy (335). In comparative clinical studies with amphotericin B, where individuals were stable or unlikely infected with fluconazole‐resistant or voriconazole-resistant pathogens, antifungal Fluconazole and voriconazole were considered effective alternatives (336–338). In cases where additional support against mold infection is required, voriconazole can be used. For SOT patients with high fever, *Candida* colonization, and other risk factors, fluconazole is favored for hemodynamically stable patients, whereas echinocandin is preferred for critically ill patients for two weeks (334). In SOT patients with kidney transplants, no therapy is required for Candiduria (no associated symptoms seen), although a ureteric stent is administered (339).

According to international guidelines, in the case of IA, Voriconazole is the first choice (340, 341). In SOT recipients, however, Voriconazole use is limited by possible drug-drug interactions mainly when immune-suppressive drugs are administered, for example, cyclosporine, tacrolimus, and sirolimus (342–345). Due to hepatotoxicity and neurological symptoms, voriconazole is often not the choice in clinical practice for early post-SOT recipients and liver transplant recipients (346). International guidelines have also recommended Isavuconazole for treating IA (340, 347). It demonstrates a lower level of toxicities in the liver, neurological symptoms, and less drug-drug interaction with tacrolimus and sirolimus, for example (348). Early on SOT recipients, particularly of liver and kidney transplants, Isavuconazole demonstrates promising potential as drug interactions and toxicities can be significantly reduced. The second line of treatment for IA consists of L-AmB (Liposomal amphotericin B). However, it is recommended in cases where treatment with triazole has been contraindicated (340, 347). SOT recipients with IA can be administered echinocandins according to the ESCMID-ECMM-ERS guidelines as a prophylaxis and conjunction infection treatment (347).

### Antifungal prophylaxis

Antifungal agent administration to all transplant recipients constitutes universal prophylaxis. A group of recipients predisposed to a higher risk of acquiring infection can be administered with targeted prophylaxis. Ideal candidate agents would have no drug-to-drug interactions and a cheap and clinically relevant efficacy and safety profile.

Randomized controlled trials for liver transplant recipients with fungal infection demonstrated that fluconazole (dose ranges from 100 to 400 mg/daily) could decrease mainly *Candida* infections (349, 350). Clinical studies have shown that amphotericin B at low dosage (1 mg/kg/day) as a targeted prophylaxis and echinocandins reduce IC efficiently (351–353). Meta-analysis has shown that the regular use of fluconazole or amphotericin B prophylaxis resulted in a decrease in IFI incidences post-liver transplantation (354). Liver transplant recipients should be preferably administered fluconazole owing to affordability, efficacy, and ease of administration (355). In the case of pancreas and kidney transplantation, fluconazole should be administered as a prophylactic if any risk factors are observed; these can be enteric drainage, vascular thrombosis, or post‐perfusion pancreatitis (356). Other prophylactic agents can be used for non‐C albicans species prevalence. Regular prophylaxis is not advised as the incidences of IC are very low after kidney transplantation. Similar is the case for heart transplant recipients (357).

The aerosolized amphotericin B lipid complex is effective prophylaxis when routinely used till 18 days post-surgery (358). Lung transplant recipients have been recommended pre-emptive therapy, whereas, for liver and heart transplant recipients, target prophylaxis is advised for IA infections (341). Most information regarding prophylaxis and pre-emptive therapy for IA is currently established from retrospective cohort and case-control studies. Support for combination therapy for IA has been demonstrated in an elaborate double-blind, placebo-controlled multi-center trial where voriconazole and anidulafungin with voriconazole monotherapy were compared (359). Combination therapy has been reported to constitute the standard treatment for invasive aspergillosis for SOT recipients across Europe (360).

### Predicting techniques, management, and follow-up of fungal infection

The key role of TDM (therapeutic drug monitoring) is establishing the therapeutic drug level that optimizes prevention and treatment and simultaneously avoids azole toxicity for clinical success. Azoles contribute to drug-drug interactions, particularly with CNI (calcineurin inhibitors). Thus, it is postulated that it is important to monitor CNI levels during the initial days of azole therapy and post-azole therapy (361). The guidelines for IFI have recommended TDM for voriconazole and posaconazole (334, 362). Many researchers have focused on evaluating Aspergillus infections where IC has not been assessed. One research has taken the data and deduced IFI in general as an outcome (357). It recommended that the plasma trough concentration be determined when azole levels are at a steady state, which in most cases is five days for voriconazole and seven days for posaconazole (363, 364). There is insufficient information to recommend regular TDM for isavuconazole.

Management of IFI is crucial as breakthrough IFI that are resistant to the antifungal drugs pose a serious limitation. Although less research data addresses the issue of breakthrough IFI, one study, in particular, has shown breakthrough IFI in recipients of lung transplants who were under antifungal prophylaxis treatment (358). IFI breakthrough of SOT recipients is attributed to numerous factors, including the local epidemiological landscape. *A. calidoustus* is the major pathogen leading to breakthrough IFI, including SOT recipients (365). Based on the impact of breakthrough IFI on high mortality and failure in treatment, it is important in an individualized approach to examine the local epidemiology, management of antifungal therapy, and clinical setting according to the recommendations (340).

In IFI, where signatures of infection are rare and unspecific, for example, in cases of IA, follow-up is mainly done on radiological response and monitoring serum GM EIA level decline. Radiological imaging should be monitored consistently until other makers demonstrate improvement. GM EIA testing in SOT recipients is also executed. However, the values for the level are yet to be confirmed. In cases of IA, reports have shown that in only one-third of SOT recipients, GM EIA tests were positive (366).

### Prevention, systemic evaluation, and recommendations- fungal infection in recipients

Guidelines by the CDC (Centers for Disease Control and Prevention) include recommendations that exposure of high-risk patients to potential fungal pathogens should be minimal. SOT recipients are advised to wear masks upon transportation in high-risk areas. Antifungal agent efficacy in high-risk conditions, for example, in hospitals with fungal infection or high levels of spores, is yet to be accurately determined. In instances where high nosocomial environmental exposure is prevalent (367), for SOT patients, it is recommended that all transplant recipients are treated with antifungal prophylaxis (368). Another preventative strategy includes microbial screening for fungal infection for both donor and recipient (369). Without highly sensitive and specific diagnostic tools, identifying invasive fungal infections (IFI) in solid organ transplant (SOT) recipients necessitates a comprehensive systemic evaluation. Each category of SOT recipient, such as heart, lung, liver, or kidney transplant recipients, exhibits varying degrees of susceptibility to infection and may be influenced by distinct risk factors.

AFS (Antifungal stewardship) in solid organ transplant application can develop advancement in antifungal regimens. Implementation of AFS in hospital policies can facilitate IFI management and treatment. The main aims and regular evaluations should be established, i.e., diagnostic strategy, antifungal type, duration, and dosage. Identification of high-risk patients and simultaneously exclusive approaches should be in place to minimize risk (370). Recommendations in clinical settings should be based on published reports and follow international guidelines. The One World One Guideline initiative presents recommendations and guidelines on the management of mucormycosis (371), rare molds (153), rare yeasts (372), and endemic mycoses (373). The local antifungal regimens can be modified as per these guidelines. The American Society of Transplantation Infectious Diseases Community of Practice also details guidelines in the context of SOT (20, 357).

### Challenges associated with present strategies- treatment interactions

It is estimated that the value of a fungal vaccine, from the concept’s inception to development and finally entering the market, is around 200-500 million dollars over ten years (374). Past decades have seen tremendous efforts in understanding the interactions between fungal infection and immune responses. However, the development of antifungal agents has been critically slow. As fungi and humans are related from an evolutionary point of view, the possibility of toxicity and limited specific fungi targets pose drawbacks. A transplant recipient will be subject to administering immunosuppressive and prophylaxis agents to avoid organ rejection and fungal infection. Results demonstrate that the cumulative effect of these agents can lead to a decrease in microbial pathogens (375). However, adversely, the interaction of the agents will affect the pharmacokinetics and toxicity, resulting in poor clinical outcomes. Antifungal agents have also been associated with drug-resistant fungal infections in transplant facilities. The transmission of airborne *Pneumocystis jirovecii* fungi from human to human can pose a threat due to drug resistance (376). Another challenge is the imbalance in the microbiota of the recipient seen in the case of hematopoietic stem-cell transplants upon administration of antibacterial, antioxoplasma, and antifungal prophylaxis. The disrupted microbiota balance can cause some fungi species or isolates to resist antifungal agents. These then can invade the blood and develop lethal invasive infections (377). Current recommended regimens are hence challenged as less susceptibility to present treatments, such as azoles and echinocandins [301], modulating the fungi’s epidemiology. It also results in the emergence of new species regarding organ transplants, for example, *C. auris* (378).

## Future perspective

### Evaluating present understanding and challenges in immune-fungal interactions in current therapies

The present understanding of immune-fungal interactions has paved the way for developing various antifungal agents. Expanding a repertoire of antifungals that can be administered independently or with prophylactic vaccines, other antifungal agents, and immune-base adjunctive therapies is crucial in immune-compromised patients and organ transplant recipients. The complete understanding of immune cells and fungal interactions in the present therapeutic setting is complicated. Early diagnosis of FI is still challenging, and tools to identify the shift of a fungal species or strain into pathogenic and invasive characteristic is yet to be seen. In the clinical setting, it is important to consider the local epidemiology, risk assessment of patients, drug-to-drug interactions, and the possibility of breakthrough infection.

### Future directions to improve recipient outcomes concerning fungal infection post-transplant

A major challenge that needs to be addressed is to evaluate with precision the effects of present immunosuppressive regimens on the emergence of particular IFIs. The antifungal properties of the immunosuppressive agent can disrupt the microbiota by promoting the selection of certain fungi species, thereby causing acquired resistance. One of the most frequently used immunosuppressive mycophenolate mofetil, having established antifungal properties, can contribute to organ transplantation to eliminate commensal and unwanted fungal species or isolates. This immunosuppressive will consequently favor the invasive and mycophenolic acid-resistant species (379), hence could lead to invasive fungal infection burden, for example, in kidney recipients (380). After validation, this treatment direction can potentially lead to the development of molecular signatures of fungal resistance to immunosuppressants, which can be used as detecting tools to forecast clinical outcomes. Furthermore, the prophylactic regimens can be orchestrated to improve the clinical outcome for transplant recipients.

Advancements in understanding, technology, and future perspective should improve the currently recommended guidelines and protocols for therapeutic prophylaxis, thereby minimizing the risks of IFIs. From a global standpoint, understanding and unraveling the local ecology can help to identify the antifungal agent in that particular environment. TDM and modifying the dose for antifungal stewardship in organ recipients. Other factors, including environmental preventive measures and chemoprophylaxis, can be anticipated in specific circumstances. More research should also focus on developing antimicrobial agents with less toxicity and drug-drug interactions. Other potential routes of drug administration should be evaluated to maintain the balance of gut microbiota. The latest technologies allow high-throughput or deep-sequencing approaches to be utilized as tools for the early detection of imbalances in the microbiota. The cumulative essence of these strategies is to enable us to move forward in developing a personalized therapeutic regimen for organ transplant recipients against IFIs.

## Concluding remarks

Clinical presentation at the earliest for IFI in SOT patients is critical yet poses challenges owing to its unspecific and heterogeneous nature. Previously, most therapies targeted adaptive immune responses; however, in the last several years, innate immune responses in organ transplants have come into the limelight. This shifting paradigm highlights the role of trained immunity in graft rejection in animal models. Nevertheless, data from human sources is pending. Hence, it is important to elucidate trained immunity in the short-term outcome of graft function and the long-term outcome of graft survival. The possibility of inhibiting innate immune response targets will promote organ survival and potentially limit the use of immune suppression in transplant patients. Immuno-modulating treatments that are efficient, safe, and pose a reduced risk of resistance have shown great potential. However, these are limited to preclinical and early clinical trials. Population-based studies have comprehensively detailed the role of various factors, including antifungal prophylaxis, in the late onset of IFI.

Furthermore, optimizing protocols for administering therapeutic prophylaxis currently in practice in clinics will significantly benefit the overall outcome of the recipients. Research work in the past decade expands our understanding of the immense diversity in fungi species and addresses challenges associated with their characterization. Yet, it is still insufficient. Hence, it is vital to develop a comprehensive knowledge of the mechanisms whereby genetic and epigenetic factors modulate traits of fungi invasiveness and their interplay immune responses in organ transplant recipients. In recent years, the development of advanced dimensionality of single-cell technologies, including cytometry by time-of-flight (CyTOF) or mass cytometry and scRNA-seq/snRNAseq have paved the way to improve our understanding regarding potential biomarkers and immune responses in organ transplant tolerance and rejection. Machine-based learning can be integrated with these technologies and provide information that can lead us to define markers of potential therapy and the progression of pathological conditions.

## Author contributions

AE: Conceptualization, Data curation, Methodology, Supervision, Writing – original draft, Writing – review and editing. HE: Writing – original draft, Writing – review and editing. AR: Writing – original draft, Writing – review and editing.
